# Animal‐borne video systems provide insight into the reproductive behavior of the Asian black bear

**DOI:** 10.1002/ece3.7722

**Published:** 2021-06-19

**Authors:** Tomoko Naganuma, Mii Tanaka, Shiori Tezuka, Sam M.J.G. Steyaert, Kahoko Tochigi, Akino Inagaki, Hiroaki Myojo, Koji Yamazaki, Shinsuke Koike

**Affiliations:** ^1^ Institute of Global Innovation Research Tokyo University of Agriculture and Technology Fuchu Japan; ^2^ Faculty of Agriculture Tokyo University of Agriculture and Technology Fuchu Japan; ^3^ Faculty of Biosciences and Aquaculture Nord University Steinkjer Norway; ^4^ United Graduate School of Agricultural Science Tokyo University of Agriculture and Technology Fuchu Japan; ^5^ Department of Forest Science Faculty of Regional Environmental Science Tokyo University of Agriculture Setagaya Japan; ^6^ Institute of Agriculture Tokyo University of Agriculture and Technology Fuchu Japan

**Keywords:** animal‐borne camera systems, mating behavior, mating system, polygamy, *Ursus* *thibetanus*

## Abstract

Previous studies on the mating system of the Asian black bear (*Ursus thibetanus*) have been limited to observations of captive populations and estimations of multiple paternities. Hence, the mating system of wild bears remains poorly understood. Animal‐borne camera systems (i.e., cameras mounted on animals) provide novel tools to study the behavior of elusive animals. Here, we used an animal‐borne video system to record the activities of wild bears during the mating season. Video camera collars were attached to four adult Asian black bears (male “A” and “B,” and female “A” and “B”) captured in Tokyo, central Japan, in May and June 2018. The collars were retrieved in July 2018, after which the video data were downloaded and analyzed in terms of bear activity and mating behavior. All the bears were found to interact with other uniquely identifiable bears for some of the time (range 9–22 days) during the deployment period (range 36–45 days), and multiple mating in males was documented. Both males and females exhibited different behaviors on social days (i.e., days when the bear interacted with conspecifics) compared with solitary days (i.e., days with no observed interactions with conspecifics). Compared with solitary days, the bears spent a lower proportion of time on foraging activities and higher proportion of time on resting activities on social days. Our results suggest that Asian black bears have a polygamous mating system, as both sexes consort and potentially mate with multiple partners during a given mating season. Furthermore, bears appeared to reduce their foraging activities on social days and engaged more in social interactions.

## Graphical abstract

Asian black bears have a polygamous mating systems, as both sexes consort and potentially mate with multiple partners during a given mating season. We documented that mating behavior affected activity budgets, with bears spending less time on feeding and more time on resting on the days when the bear interacted with conspecifics. Animal‐borne video systems are useful to reveal the behavior of elusive species.

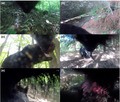

## 1 INTRODUCTION

The mating system of an animal population or species refers to the general behavioral strategies that are used by males and females to obtain mates, and include mate acquisition and the number of mates obtained by an individual (Emlen & Oring, 1977). Mating systems and strategies across animals are driven by parental care, social and ecological environment, and the strength of sexual selection, which is determined by male–male competition for females and female mate choice (Shuster, 2009; Shuster & Wade, 2003). Sexual selection has resulted in pronounced sexual size dimorphism and the evolution of polygamous mating systems in a large number of mammals (Andersson & Iwasa, 1996). Knowledge about mating systems not only has ethological values, but also is important for species‐specific conservation and management, as well as biodiversity conservation in general (Palomero et al., 2007). However, a profound understanding of mating systems currently exists only for a relatively small number of animal species (Arnold & Duvall, 1994; Steyaert et al., 2020), as mating system research is challenging, especially for elusive species.

Among bears (Ursidae), mating behavior and mating systems are probably best documented for polar bears (*Ursus maritimus*), brown bears (*U*. *arctos*), and American black bears (*U*. *americanus*). Their mating activities occur in early summer, and they are considered as polygamous in the broadest sense, as both males and females mate with multiple members of the opposite sex during a given mating season (Spady et al., 2007; Steyaert et al., 2012). However, it is also known that bear populations and species exhibit variation in their mating systems, strategies, and behaviors, despite sharing several characteristics (e.g., sexual size dimorphism, polygamy) (Spady et al., 2007).

Knowledge about the mating behavior of the Asian black bear (*U*. *thibetanus*) in the wild is limited (Steyaert et al., 2020). As it is an elusive species, making direct observations in natural habitat is difficult, and a lack of intensive research limits our knowledge. However, adult aggregations and mating behavior have been observed in wild bears (Hashimoto & Anrui, 2010). Both sexes can mate various times and with various partners in captivity (Yamamoto et al., 1998), and multiple paternity litters can occur in the wild (Yamamoto et al., 2013). This indicates that Asian black bears have a seasonal polygamous mating system. However, little information is available on the mating system of wild bears and no detailed observations of their mating behavior have been reported to date.

Biologging involves the attachment of sensors or tracking devices to animals and enables many aspects of the life histories of both terrestrial and marine species to be documented or measured (Hussey et al., 2015; Kays et al., 2015; Wilmers et al., 2015). Despite the huge technological advances in terms of biologging, determining the exact behavior of an animal at a given point in time remains difficult with traditional (high‐resolution) global positioning system (GPS) tracking devices, accelerometers, or various physiological sensors, as ground‐truthing data typically lack. However, recent advances in camera technologies have increased the potential for recording georeferenced animal behavior at any given point in time (Thompson et al., 2012). Such cameras are typically programmed to collect behavioral data for short time intervals (“clips”). For bears, these behaviors include stalking prey and predation outcome, sleeping, and mating, as well as search and handling time of food items (Bowersock et al., 2015; Brockman et al., 2017).

Here, we used this new camera technology to record the mating behavior of wild Asian black bears. We deployed collars with an animal‐borne video camera on a small number of wild bears that were known to be sexually mature to shed light on this species’ behavioral mating systems, which was for the primary objective of this study defined as interactions with other bears, especially of the opposite sex. By evaluating social interactions and mating behavior between the camera‐equipped bears and unmarked individuals, we expected that 1) Asian black bears in the wild are indeed polygamous, which was shown in captive bears (Yamamoto et al., 1998). Given that, in brown bears, breeding bears devoted smaller proportion of their time to feeding, compared with nonbreeding bears (Fernández‐Gil et al., 2006), we predicted that 2) Asian black bears may trade foraging activities for mating related behavior when together with a conspecific of the opposite sex. Here, we also evaluated these two general aspects of animal mating systems to demonstrate the potential of these new camera systems in studying the mating behavior of elusive species. We discuss limitations and strengths of this novel technology to evaluate the mating behavior of wild bears, and compare our results with those of previous studies.

## 2 MATERIALS AND METHODS

### 2.1 Study area

The study was conducted in Okutama Town (35°48′N, 139°5′E), which is approximately 100 km west of the Tokyo Metropolis. This region has heavy rainfall in summer and little snow in winter. In the period 2006–2017, the mean annual precipitation was 1,510 mm and the mean annual temperature was 12.4℃ (range: 0.6℃ in January to 24.2℃ in August) (Japan Meteorological Agency, 2020).

Conifer plantations consisting of Japanese cedar (*Cryptomeria* *japonica*), Japanese cypress (*Chamaecyparis obtusa*), and Japanese larch (*Larix kaempferi*) cover approximately 50% of the study area, whereas natural forests dominated by *Castanea crenata* and *Quercus serrata* in the lower mountain zone [400–500 m above sea level (a.s.l.)], *Q*. *crispula*, *C*. *crenata*, and *Fagus crenata* in the middle zone (500–1,500 m a.s.l.), and *Abies homolepis* and *Tsuga diversifolia* in the upper zone (1,500–1,800 m a.s.l.) cover about 40% of the area (Koike et al., 2008). The remaining area comprises infrastructure and agriculture land.

### 2.2 Bear capture

Asian black bears were captured from May to June 2018 using barrel traps baited with honey. A total of five traps were set within 5 km of each other, and the trapped bears were immobilized with tiletamine hydrochloride and zolazepam hydrochloride (Virbac, Carros, France, 8 mg/1kg of body weight). Basic body measurements were taken, and premolar extraction was performed for age determination. Bears over 40 kg were equipped with microchips for individual identification and GPS/camera collars (GPS Vertex Lite Collars; Vectronic Aerospace GmbH, Berlin, Germany). The collars (1.2 kg) weight less than 3% of body mass of bears. Bear capture and handling were approved by the Institutional Animal Care and Use Committee of Tokyo University of Agriculture and Technology. All experimental procedures used in the bear research followed the Guidelines Concerning Animal Experimentation of the Tokyo University of Agriculture and Technology and the Mammal Society of Japan (Mammal Society of Japan 2020).

### 2.3 Camera schedules

As Asian black bears in the study area are mostly diurnal (Yamazaki et al., 2008), we programmed the collars to record a 10‐s video clip of the bear every 15 min, with a duty cycle of 13 hr on (5:00–17:45; total 52 clips per day) and 11 hr off, based on sunset/sunrise times in June and July in the study area. Video recording started at 5:00 on the day after capture. Each camera had a battery life of about 18h, and video data were stored onboard the devices. Since captive bears mostly exhibit mating behavior between June and July (Yamamoto et al., 1998), the battery life and onboard memory capacity of the camera‐collar technology were considered sufficient for the collection of frequent observations of mating behavior. The collars were dropped by remote control at the end of the programmed lives of the camera batteries. The collars were then recovered and returned to the manufacturer for downloading the video clips in the mp4 format.

### 2.4 Behavioral data analysis

To avoid potential effects of handling and attempted remote collar drop‐offs, only the data collected between 5:00 on the day after capture and 17:45 on the day before their collars drop‐off were used in the analyses. Each video clip was first assessed for the presence of other bears in the recording. However, it is possible that the number of interactions with other bears may be undercounted if the other bear was not directly in front of the camera or the camera view was obscured by hair or soil. To address this uncertainty, any day during which a conspecific was captured on video at least once was considered to be a day in which the bear interacted with other bears. Using this definition, the clips were classified into two groups: “social days” (i.e., days when the bear interacted with conspecifics) and “solitary days” (i.e., days with no observed interactions). Each video clip was then assigned a specific behavior: “traveling,” “sleeping,” “resting,” “foraging,” “sniffing,” “others,” and “unclear” (7 behaviors in total). For the ethogram used in this study, see Table 1. Clips from social days were further classified according to the behavior of the conspecific.

**TABLE 1:** Ethogram of behavioral classes of Asian black bears distinguished on the camera‐collar recording

Behavior	Description
Traveling	Movement on land such as running or walking
Sleeping	No movement; bear is completely inactive
Resting	No movement; bear is grooming, standing, and lying down. Not sleeping.
Sniffing	Sniffing something excluding food items
Foraging	Actual feeding behaviors and behaviors of searching for foods (i.e., digging ground, breaking decayed tree, stripping tree bark, sniffing or touching food items, and moving or breaking branches on the tree with food items)
Others	Social engagement (i.e., touching body parts of another bear), mating, drinking, bedding, and moving on trees without food items
Unclear	The video could not be determined due to issues such as hair covering the camera.

Whenever possible, videoed conspecifics were assigned a sex and uniquely identified by multiple researchers based on external characteristics such as external genitalia, facial scars, and the shape of the white patch on their breast. Individual recognition followed research, which has been used in brown bear studies (Clapham et al., 2012; Nawaz et al., 2008; Shimozuru et al., 2017). Most Asian black bears have a characteristic white patch on their chest (e.g., large bib‐like mark, small point‐like mark, or slim line mark), the size and shape of which is characteristic of the individual. Identification using these chest marks is confirmed to be highly reliable in this species (Higashide et al., 2012). Bears that could not be identified were considered as “unknown individuals,” while those for which the sex could not be clearly identified were classified “unknown sex.” Bears for which the researchers could not agree on an identification were also considered as unknown individuals. If bear mating was recorded by a male bear's camera, the other bear was assumed to be female. After the behavioral classification, the daily proportion of each behavior was calculated for each social and solitary day and for each bear (Table 2). We analyzed the video data using Microsoft Movies and TV and Windows Media Player.

**TABLE 2:** Mean proportion of time spent on each activity (%) on “social days” and “solitary days”

	Male A		Male B		Female A		Female B	
	Solitary days	Social days	Solitary days	Social days	Solitary days	Social days	Solitary days	Social days
Traveling	19.0 ± 12.2	24.0 ± 17.8**	15.3 ± 6.1	12.8 ± 11.3	5.5 ± 5.8	5.5 ± 9.6	14.6 ± 8.8	8.3 ± 9.3*
Sleeping	23.0 ± 15.5	19.8 ± 11.8	22.5 ± 17.0	29.8 ± 18.7**	47.8 ± 26.6	38.3 ± 24.0*	33.0 ± 13.3	41.2 ± 18.9**
Resting	7.9 ± 4.4	15.6 ± 13.3**	6.6 ± 4.6	14.5 ± 9.3**	3.7 ± 1.6	10.6 ± 6.5**	10.3 ± 5.4	14.8 ± 7.7**
Sniffing	17.1 ± 8.7	17.2 ± 10.7	10.1 ± 7.3	8.0 ± 5.8	9.9 ± 5.5	7.7 ± 6.4	15.8 ± 8.3	9.1 ± 8.9*
Foraging	16.4 ± 11.8	6.0 ± 5.5*	27.8 ± 23.5	18.7 ± 21.0*	9.7 ± 8.5	4.6 ± 6.0*	15.5 ± 12.5	9.4 ± 12.7*
Others	0.4 ± 0.9	1.9 ± 1.7**	0.5 ± 1.3	3.0 ± 3.1**	0.6 ± 1.2	1.6 ± 2.1**	2.1 ± 1.9	2.6 ± 2.0
Unclear	16.0 ± 13.0	15.4 ± 11.5	17.1 ± 15.9	13.1 ± 9.1*	22.7 ± 15.2	31.8 ± 22.8**	8.6 ± 8.5	14.6 ± 10.7**

* Significantly decreased compared with solitary days

** significantly increased compared with solitary days

### 2.5 Statistical analysis

Permutational multivariate analysis of variance (PERMANOVA) was used to test whether bears exhibited behavioral changes in response to having a social day or a solitary day. The presence or absence (0, 1) of each behavior per bear was used as response variables in relation to bears having a social day or a solitary day. The Jaccard distance, which is the dissimilarity matrix quantified directly from count data, was calculated to be used in the PERMANOVA based on 1,000 permutations. Significance testing for differences in occurrence of each behavior by social days or solitary days was performed using Fisher's exact contingency test (Fisher, 1935). The analysis was conducted using the “adonis” function in the “vegan” package (Oksanen et al., 2020) and “fisher.bintest” function in the “RVAideMemoire” package (Hervé 2020) of the statistical software R 3.5.3 (R Core Team 2020) with a significance level of 0.05 based on the adjusted p‐value for multiple comparisons.

## 3 RESULTS

### 3.1 Bear tracking

In total, four Asian black bears were equipped with camera collars: two 4‐year‐old males (male A, 48 kg; and male B, 42 kg), a 12‐year‐old female (female A, 52 kg), and a 4‐year‐old female (female B, 42 kg). Video data from each bear were analyzed for the following periods: male A, 25 May–8 July (45 days, 2,326 clips in total); male B, 4 June–17 July (44 days, 2,273 clips); female A, 11 June–16 July (36 days, 1857 clips); and female B, 1 June–10 July (40 days, 2074 clips). The total number of clips per bear was less than the theoretical maximum number of clips for the observation period because the cameras occasionally malfunctioned.

**FIGURE 1:**
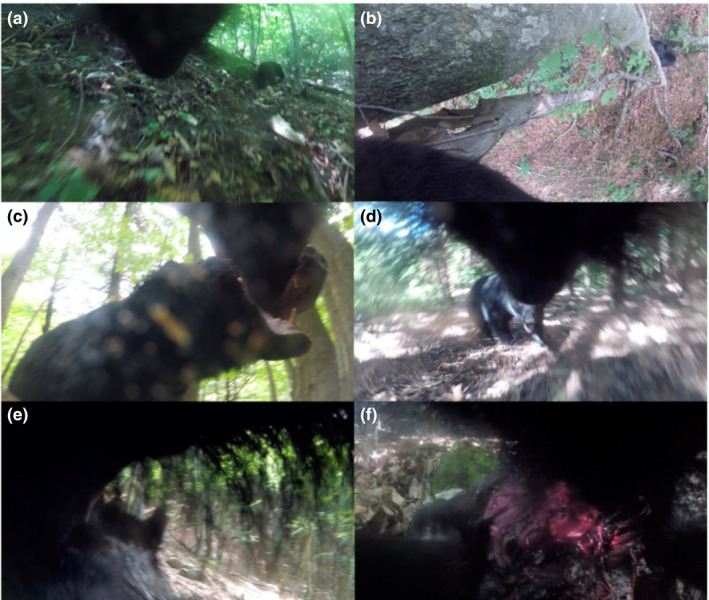
Examples of images taken from video clips recorded during mating season in 2018. (a) Video still of male B pursuing female 2. (b) Video still of male 1 guarding female A, who was resting in a tree. (c) Video still of male 1 as seen by female A. Both individuals have their mouths open and are believed to be trying to communicate in some way. (d) Video still of male B meeting male 1. (e) Image of male B mating with female 3. Female 3’s ear can be seen in front of male B. (f) Video still of male B feeding on a bear cub

A total of 477 clips recorded a conspecific (Figure 1), of which 458 clips (male A: 30 clips, male B: 139 clips, female A: 113 clips, and female B: 176 clips) recorded identified individuals (i.e., unmarked female and male, and unknown sex) and 19 clips recorded unknown individuals (male A: 2 clips, male B: 12 clips, female A: 3 clips, and female B: 2 clips) (refer to Appendix 1 for detailed video examples).

### 3.2 Behavioral differences between social and solitary days

The ratio of social days to solitary days for each bear was as follows: male A, 9/36 (20.0% social days); male B, 19/25 (43.2% social days); female A, 22/14 (61.1% social days); and female B, 21/19 (52.5% social days). The results of PERMANOVA showed significant effects of social days/solitary days on the behavior in all bears (male A: *p* < .001, male B: *p* < .001, female A: *p* < .001, and female B: *p* < .001), which imply that all bears exhibited significant differences in their behavior between social and solitary days. Compared with solitary days, all bears rested more and foraged less on social days (Table 2). The differences in other behaviors between social and solitary days varied among individuals (Table 2).

### 3.3 Individual mating behavior

Male A interacted with two other unique bears during the observation period (Table 3). One of those bears was a female (unmarked female 1), with whom male A interacted for two days and exhibited mating behavior on two days (16:30 on 30 May and 9:30 on 2 June). Male A then interacted with a different bear of which the sex could not be identified (unknown sex 1) from 4 July to 8 July.

**TABLE 3:** Timelines of social interactions of bears in Okutama, Japan, during mating season in 2018. “x” are indicated in “solitary days,” and the bears that interacted with the collard bear are indicated in “social days” (F1: female 1, F2: female 2, F3: female 3, M1: male 1, M2: male 2, M3: male 3, M4: male 4, UnS1: unknown sex 1, UnS2: unknown sex 2, UnS3: unknown sex 3, and U: unknown individual)

Day	Male A	Male B	Female A	Female B
25 May	x			
26 May	x			
27 May	x			
28 May	x			
29 May	x			
30 May	F1*			
31 May	x			
1 Jun	x			**U**
2 Jun	F1*			x
3 Jun	x			x
4 Jun	x	x		x
5 Jun	x	x		x
6 Jun	x	x		x
7 Jun	x	x		x
8 Jun	**U**	x		**UnS3**
9 Jun	**U**	**U**		**UnS3**
10 Jun	x	x		x
11 Jun	x	x	**U**	x
12 Jun	x	**UnS2**	x	x
13 Jun	x	**UnS2**	x	x
14 Jun	x	**UnS2**	**U**	x
15 Jun	x	x	x	x
16 Jun	x	**U**	x	x
17 Jun	x	x	**M2**	x
18 Jun	x	**U**	**M2**	x
19 Jun	x	x: cub**	**M2**	x
20 Jun	x	**U**	**M2**	x
21 Jun	x	x	**M2**	**U**
22 Jun	x	x	**M2**	x
23 Jun	x	**U**	**M2**	x
24 Jun	x	x	**M2**	**M3**
25 Jun	x	x	**M2**	**M3**
26 Jun	x	x	**M2**	**M3**
27 Jun	x	**U**	**M2**	**M3**
28 Jun	x	x	**M2**	**M3**
29 Jun	x	x	**M2**	**M3**
30 Jun	x	**F2**	x	**M3**
1 Jul	x	F2*	x	**M3**
2 Jul	x	M1***, F2*	x	**M3**
3 Jul	x	**F2**	x	**M3**
4 Jul	**UnS1**	x	x	**M3**
5 Jul	**UnS1**	x	x	**M3**
6 Jul	**UnS1**	x	**M2**	**M4**
7 Jul	**UnS1**	**U**	**M2**	**M4**
8 Jul	**UnS1**	x	**M2**	**M4**
9 Jul		x	x	**M4**
10 Jul		x	x	**M4**
11 Jul		x	x	
12 Jul		F3*	x	
13 Jul		**F3**	**U**	
14 Jul		F3*	**M2**	
15 Jul		F3*	**M2**	
16 Jul		F3*	**M2**	
17 Jul		x		

* Mating, ^**^Feeding, and ^***^Fighting.

Male B interacted with three other bears during the observation period, two of which were female (females 2 and 3) (Table 3). Male B first interacted with a bear of unknown sex (unknown sex 2) from 12 to 14 June. On 19 June (7:00), a clip of the male foraging on a conspecific was recorded, which, based on the body size, was likely a cub of the year. Before this event, clips of male B traveling (5:30, 5:45, and 6:45), sniffing (6:00 and 6:15), and foraging on understory herbs (6:30) were recorded, although no clips of interactions with the cub's mother were recorded. Male B then interacted with female 2 for 4 days from 30 June to 3 July. On the second day (1 July), mating between male B and female 2 was recorded for the first time (9:00). On the third day (2 July), after resting near female 2 on a tree (14:15), fighting between male B and another male (male 1) on the ground was recorded (14:30): Male B walked toward male 1, which stayed still while looking at male B; the two males then fought while standing on their hind legs, after which male 1 ran back away from male B. On the same day, two clips of mating between male B and female 2 were recorded (16:00 and 17:30). Finally, male B interacted with female 3 for five days (12–16 July). During this time, mating between male B and female 3 was recorded in two clips taken on 12 July (17:30 and 17:45), two clips taken on 14 July (14:30 and 14:45), two clips taken on 15 July (5:45 and 6:00), and two clips taken on 16 July (15:15 and 15:30).

Female A interacted with two other bears during the observation period, of which one was a male (male 2) (Table 3). Female A interacted with male 2 during three separate occasions that lasted multiple days, that is, 13 days (17–29 June), and two times for 3 days (6–8 July and 14–16 July).

Female B interacted with three other bears for observation period, of which two were male (males 3 and 4) (Table 3). The sex of the third bear could not be identified (unknown sex 3), and this bear was recorded from 8 to 9 June. Female B interacted with male 3 for 12 days (24 June – 5 July) and later with male 4 for 5 days (6–10 July). On 5 July, female B rested on a tree close to male 3 (6:00) before the two moved closer (7:00). After that, female B and male 3 traveled together until the last record of male 3 was made (15:45–16:15, and 16:45). On 6 July, the first record of male 4 was made: The male stayed close to female B, while the female sniffed the ground (13:30) and groomed itself (13:45).

## 4 DISCUSSION

Using the new technology of animal‐borne video recording revealed novel insight into the mating behavior and system of the Asian black bear, despite the low sample size of only four study units. Our data support that Asian black bears in the wild have a polygamous mating system (Yamamoto et al., 2013), as individuals of both sexes consorted and potentially mated with various partners during the mating season. In addition, we documented that Asian black bears express different activity budgets on social days, compared with solitary days. Specifically, compared with solitary days, they appeared to spend less time foraging and more time resting on social days, but behavioral adjustments were ambiguous and differed between individuals.

Brown bears have a polygamous mating system in the broadest sense, as both sexes can associate and mate with various partners during a mating season (Steyaert et al., 2012). Our study shows that in Asian black bears, individuals of both sexes can interact (i.e., an indication for mating behavior in an otherwise solitary species) with more than one individual of the opposite sex during the mating season, and we documented multiple mating in males. Those observations suggest that Asian black bears have a polygamous mating system. However, it remains unclear whether females actually mated with the males they associated with, due to the camera orientation on the female's collar and the mating position of bears (males mounting females). Additional observations obtained from animal‐borne video systems and from a larger number of individuals of both sexes can advance our knowledge about the mating system and mating strategies in wild Asian black bears.

Many species, including bears, experience lactational anestrus. In such species, male infanticide can be common as male reproductive strategy (i.e., sexually selected infanticide). When litter loss occurs during the mating season, the victimized mothers are released from lactation and can enter estrus and mate shortly (within days) after the loss (Steyaert et al., 2014). Hence, males can create mating opportunities and increase their reproductive success by killing entire litters of unrelated offspring during the mating season (Bellemain et al., 2006; Bellemain, Zedrosser, et al., 2006; Steyaert et al., 2014; Swenson et al., 2001). Collecting infanticide cases in wild bears is extremely difficult, and until now, only anecdotical evidence of infanticide in Asian black bears exists. However, our camera collars recorded an adult male bear consuming a cub of the year, which might have been an infanticide victim. Unfortunately, our data cannot provide information about how the male obtained the bear cub because no clips of interactions between the adult male and the cub's mother were recorded. The camera collars can be helpful for detecting potential infanticide cases. Combined with field investigations and genetic tools (Bellemain, Swenson, et al., 2006; Bellemain, Zedrosser, et al., 2006), this technology is promising for shedding light on the functional significance of infanticide in Asian black bears.

The energy balance is an essential factor to evaluate the nutritional state of bears (Robbins et al., 2004). The energy balance of Asian black bears has previously been suggested to follow a seasonal, bimodal pattern, dropping from spring to summer and increasing from summer to a peak in autumn (Furusaka et al., 2019). In particular, decreased food consumption by Asian black bears in summer (i.e., June and July) was associated with a large decrease in their energy balance. One factor that has been proposed to explain this is low availability of food resources during this time of the year (Fuchs et al., 2019; Furusaka et al., 2019). However, our results suggest that within this period, bears decreased foraging activities on social days compared with solitary days, which accounted for about half of the days in the mating season in one male and both females. This implies that not only reduced forage availability, but also mating‐induced reduced foraging may explain a general decrease in their energy balance during this period of the year.

This decrease in foraging activity concurrent with mating activities has been suggested in brown bears (Fernández‐Gil et al., 2006) and may support our observations. In addition, male brown bears and American black bears have also been observed consorting with specific females for prolonged periods of time and can have exclusive partnerships with specific females (Spady et al., 2007; Steyaert et al., 2012). Such mating behavior was also observed in the present study, suggesting that mate guarding is part of the mating system of Asian black bears. Mate guarding also suggested to constrain feeding activity in male primates (Alberts et al., 1996; Georgiev et al., 2014; Matsubara, 2003). Therefore, several behaviors associated with mating seem to induce decreased foraging activity for male bears during the mating season. Although behavioral changes related to sexual selection are mostly expected to occur in males (Clutton‐Brock & McAuliffe, 2009), the same or similar behavioral changes within both sexes (i.e., the decrease in foraging and increase in resting when being social) were observed in the present study. However, our study only involved four focal study units, which implies that the interpretation of our behavioral observations is suggestive and has to be viewed with caution.

Male–male competition for access to receptive females based on the outcomes of aggressive behavior and fights has been shown in brown bears (Steyaert et al., 2020). In the present study, the camera collar recorded that the focal adult male fought with an unmarked male and appeared to win the contest as the same unmarked male backed off. Despite no female being recorded in the clip of this fighting, the focal male interacted and mated with same female before and after the fight, which suggests that the male successfully guarded the female in this occasion. To our knowledge, this was the first direct observation of contest competition for mates in male Asian black bears. However, fighting behavior was not observed in other cases when males or females changed partners.

The Asian black bear is distributed from West to East Asia. However, poaching combined with habitat declines has reduced most local populations across Asia, and the Asian black bear is currently listed as vulnerable on the Red List by the International Union for Conversation of Nature (Garshelis & Steinmetz, 2020). Despite the small sample size, we documented ecologically important behaviors of free‐ranging Asian black bears. This indicates that animal‐borne camera systems can contribute to a better understanding of the behavior of this species. Therefore, this technology could accelerate research on Asian black bear ecology about which particularly little is currently known. This could include behavioral responses toward conspecifics, humans, or human activities, or the identification of key habitat features within their home range. As such, camera collars have the potential to aid the conservation of larger mammals.

Despite having provided behavioral data that would have been extremely difficult or even impossible to gather using other methods, camera collars currently have several limitations in their use. First, identification of conspecifics appearing in the video footage remains difficult. The footage used in this study was sufficiently clear to allow detection of other bears near the focal bear, but there were many cases where the distances between the focal bear and the other bears were unclear, and many bears could not be identified or their sex determined. Second, we used 10‐s recordings every 15 min during daytime, meaning that the animals’ behaviors at night and during the time windows between recordings remain unknown. Third, females had many more unclear behaviors during the mating season than males. For example, it was only possible to determine whether a male was chasing a female if the female turned back to look, and it was difficult to determine whether mating took place based on the footage obtained from the cameras fitted on females. Thus, given the current configuration of the camera collars, it may be more practical to primarily outfit males with the cameras and use ear tags for identifying females. In addition, the cameras provided only visual information. Olfaction is a particularly important mode of communication for various terrestrial mammals, including solitary carnivores. In the obtained video clips, the bears appeared to sniff the ground, trees, and air, which implies that they had encountered olfactory cues of conspecifics, even if other bears were not observed in the clips. Despite these limitations, the use of camera collars allowed us to shed light on important aspects of the mating behavior of the Asian black bear. Therefore, these collars hold promise as a tool to help better understanding the ecology of Asian black bears and other large and elusive mammals.

## 5 CONCLUSION

Very little information about the mating system of the Asian black bear in the wild currently exists, in part because it is an elusive species, making systematic observations in the wild difficult. Animal‐borne video systems can be a powerful tool to shed light on the behavior of such elusive species. Despite a very low sample size (two males and two females), our study advanced our current knowledge on the mating system of the Asian black bear. First, our observations suggest that wild Asian black bears are polygamous, as all bears interacted with more than one individual of the opposite sex, and we documented multiple mating in males during the mating season. Second, we documented that mating behavior affected activity budgets, with bears spending less time on feeding and more time on resting on social days. Such changes may add to the cost of reproduction the species experiences. In addition, we recorded contest competition among males for females and a potential infanticide case. Infanticide can be an important part of the mating system of polygamous species (sexually selected infanticide) and is extremely difficult to witness in the wild. Improving our knowledge on the mating system of the Asian black bear will contribute to improved management and conservation of the species.

## CONFLICT OF INTEREST

The authors declare that they have no competing interests.

## AUTHOR CONTRIBUTIONS

**Tomoko Naganuma:** Conceptualization (equal); Data curation (lead); Formal analysis (equal); Funding acquisition (supporting); Investigation (equal); Writing‐original draft (equal); Writing‐review & editing (lead). **Mii Tanaka:** Data curation (equal); Formal analysis (equal); Investigation (equal); Writing‐original draft (supporting). **Shiori Tezuka:** Data curation (equal); Formal analysis (equal); Investigation (equal); Writing‐original draft (supporting). **Sam M.J.G. Steyaert:** Conceptualization (equal); Writing‐original draft (equal); Writing‐review & editing (equal). **Kahoko Tochigi:** Conceptualization (supporting); Data curation (equal); Formal analysis (lead); Investigation (equal); Writing‐original draft (equal). **Akino Inagaki:** Conceptualization (supporting); Data curation (equal); Investigation (equal). **Hiroaki** **Myojo:** Conceptualization (equal); Data curation (equal); Investigation (lead). **Koji Yamazaki:** Conceptualization (equal); Funding acquisition (equal); Investigation (lead); Project administration (lead); Supervision (lead); Writing‐review & editing (equal). **Shinsuke Koike:** Conceptualization (lead); Funding acquisition (equal); Investigation (equal); Project administration (equal); Supervision (lead); Writing‐original draft (equal); Writing‐review & editing (equal).

## Supporting information

Video S1

## Data Availability

All data in this manuscript are available on Dryad (https://doi.org/10.5061/dryad.t76hdr814).

## APPENDIX 1

Examples taken from video clips recorded during mating season in 2018. The video can be accessed through the URL https://youtu.be/pKIXH4mrejc.
